# Antifungal-Loaded Calcium Sulfate Beads as a Potential Therapeutic in Combating Candida auris

**DOI:** 10.1128/AAC.01713-21

**Published:** 2022-01-18

**Authors:** Mark C. Butcher, Jason L. Brown, Donald Hansom, Rebecca Wilson-Van Os, Craig Delury, Phillip A. Laycock, Gordon Ramage

**Affiliations:** a Oral Sciences Research Group, Glasgow Dental School, School of Medicine, Dentistry and Nursing, College of Medical, Veterinary and Life Sciences, University of Glasgow, Glasgow, UK; b Forth Valley Royal Hospital, Larbert, UK; c Biocomposites Ltd., Keele, Staffordshire, UK

**Keywords:** biofilm, fungal, wound management, antimicrobial, *Candida auris*, antimicrobial activity, calcium sulfate

## Abstract

Candida auris provides a substantial global nosocomial threat clinically. With the recent emergence that the organism can readily colonize skin niches, it will likely continue to pose a risk in health care units, particularly to patients undergoing surgery. The purpose of this study was to investigate the efficacy of antifungal-loaded calcium sulfate (CS) beads in combatting C. auris infection. We demonstrate that the CS-packed beads have the potential to interfere with planktonic and sessile C. auris.

## INTRODUCTION

Candida auris is a nosocomial pathogen that was first identified in 2009 (1). The organism has been subject to extensive scrutiny in the field of medical mycology over the past decade, primarily due to the organism’s multidrug resistance (2). A notable pathogenic trait of C. auris is its ability to persist in the environment, surviving on abiotic surfaces for up to weeks via the formation of biofilms (3). In addition to this, the fungus has also been shown to readily inhabit skin, forming high-burden biofilms when grown in artificial sweat on a porcine skin model (4). *In vivo*, in a murine model, C. auris establishes residence on the skin tissue (5), while a clinical study has highlighted that the organism can coexist among microbiota and other mycobiota within the skin flora (6). Thus, C. auris infection could provide a substantial infection risk to those patients with open wounds in intensive care settings.

The use of calcium sulfate (CS) as a biomaterial is not uncommon. CS has been developed as a vehicle for delivering antibiotics to infected surgical sites (7). The mechanism of action for such a biomaterial involves the slow dissolution of the CS at the infection site, resulting in a steady elution of the loaded drugs locally into the immediate microenvironment. Several studies have highlighted the efficacy of using loaded CS beads in knee and hip periprosthetic joint infections (reviewed in reference 8), while their use in treatment of diseases, such as diabetic foot ulcers and osteomyelitis, is starting to be explored (9, 10).

This study serves to investigate the potential use of antifungal-loaded CS beads in preventing C. auris biofilm formation and associated pathogenicity. The efficacies of three antifungals, caspofungin (CSP), fluconazole (FLZ), and amphotericin B (AMB) incorporated into CS beads, were tested against planktonic and sessile C. auris NCPF 8978. The potency of the antifungal-loaded CS beads would also be assessed in a three-dimensional (3D) organotypic skin epidermis model.

First, a broth microdilution test was used to assess drug release across the 7 days of suspension of CS beads in RPMI media. C. auris was found to be susceptible to a released concentration of approximately 1.2 μg/mL for CSP across all days, varied from 40 to 80 μg/mL for FLZ, and maintained at 0.9 μg/mL for AMB over the full 7 days. Specifically, the concentration of FLZ was 80 μg/mL on days 1, 5, 6, and 7, while 40 μg/mL on days 2, 3, and 4.

**FIG 1:**
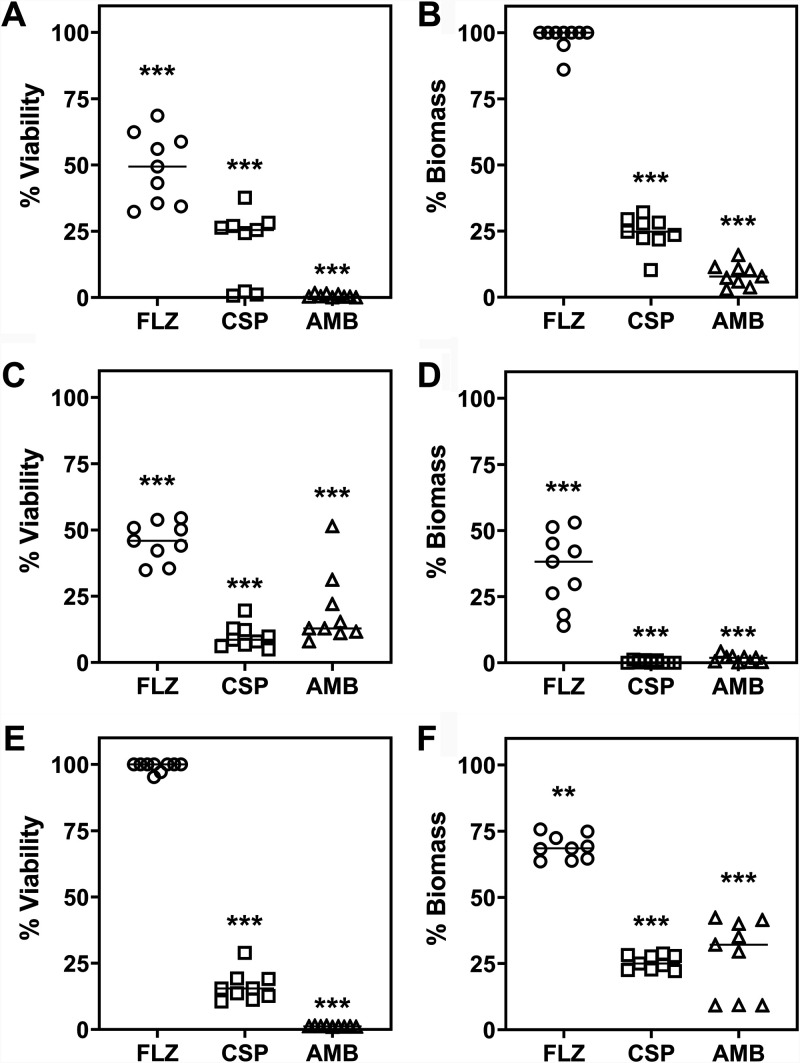
Inhibition of sessile cell viability and fungal biomass by antifungal CS beads. Biofilms were treated with FLZ (80 mg)-, CSP (35 mg)-, and AMB (25 mg)-loaded antifungal beads for 1, 3, and 5 days. The XTT and CV assays were used to assess metabolic activity and biofilm biomass, respectively. Results shown as % viability (A, C, and E) and % biomass (B, D, and F) relative to CS-treated biofilms minus the antifungal. Significant reductions shown as follows: **, *P* < 0.01; ***, *P* < 0.001.

For the biofilm studies, results of the XTT [2,3-bis-(2-methoxy-4-nitro-5-sulfophenyl)-2H-tetrazolium-5-carboxanilide salt] and crystal violet (CV) assays indicated that CSP and AMB were more effective than FLZ in reducing the metabolic activity and biomass of the C. auris biofilms across all three time points (***, *P* < 0.001) (Fig. 1A, C, and E). Interestingly, FLZ-treated biofilms displayed reduced viability after 1- and 3-day treatments (Fig. 1A and C) but were completely metabolically active following treatment for 5 days with the antifungal (Fig. 1E). Biofilms exposed to FLZ beads revealed little to no biomass alteration compared with control CS beads at day 1 (Fig. 1B) but significant reductions after 3- and 5-day treatments (**, *P* < 0.001; ***, *P* < 0.001) (Fig. 1D and F). CSP and AMB treatment significantly reduced the biomass of the biofilms over the entire time course of the experiment (all ***, *P* < 0.001, respectively).

**FIG 2:**
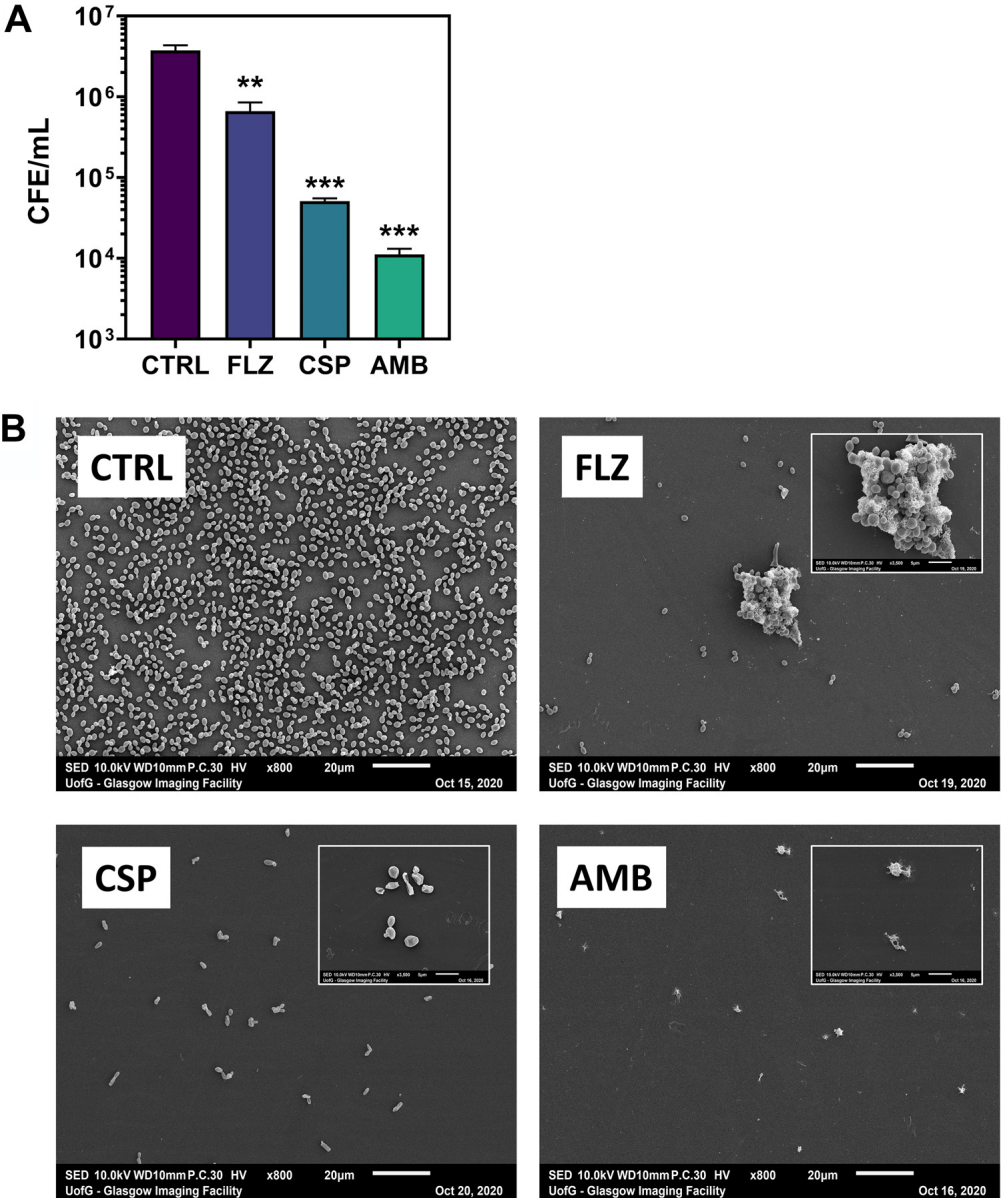
Molecular and microscopic assessment of C. auris biofilm inhibition. Biofilms grown on 13-mm Thermanox coverslips were treated with FLZ (80 mg)-, CSP (35 mg)-, and AMB (25 mg)-loaded antifungal beads for 3 days. (A) Following treatment, DNA was extracted and colony forming equivalents determined using qPCR. (B) Scanning electron microscopic images highlight clear reductions in the biofilm bioburden. Significant reductions shown as follows: **, *P* < 0.01; ***, *P* < 0.001.

Given that 3-day treatment gave the most potent effect with the antifungal against C. auris (Fig. 1), biofilms were grown for 24-h and treated with each antifungal for 3 days. Then, quantitative PCR (qPCR) assessment and scanning electron microscopy (SEM) imaging was used to assess the C. auris biofilm inhibition. Results from the qPCR corroborated the above observations in that FLZ treatment was the least effective of the three antifungals in reducing the fungal load, although this was still significantly lower than that of the control CS bead-treated biofilms (**, *P* < 0.01) (Fig. 2A). SEM imaging of the treated biofilms showed a densely populated C. auris biofilm treated with CS beads only. FLZ-, CSP- and AMB-treated biofilms were much more sparsely populated with yeast cells. These results indicate that all three antifungals were effective in reducing the fungal load of the biofilms after 3 days of treatment with antifungal-loaded CS beads.

**FIG 3:**
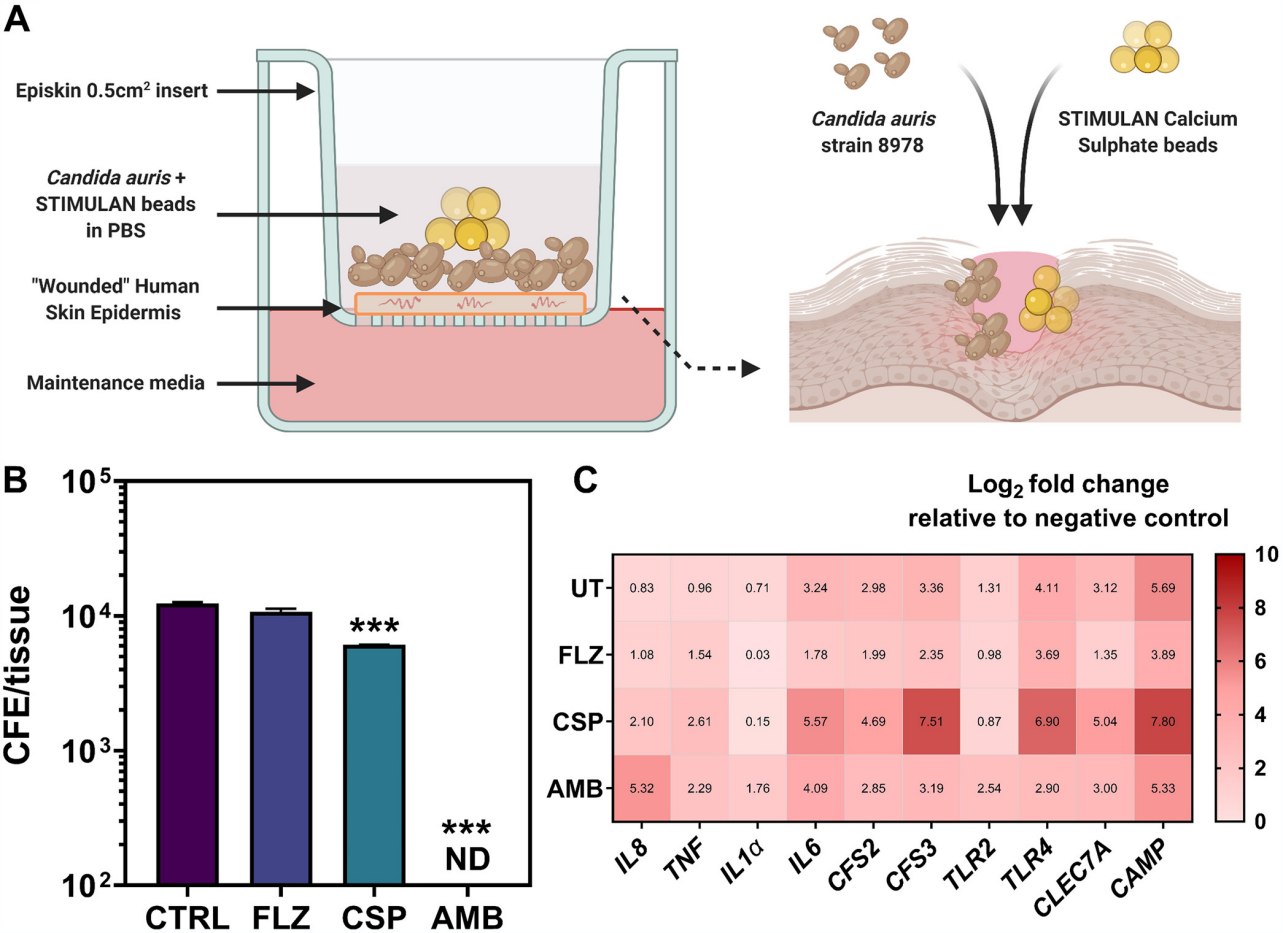
Assessing the efficacy of the antifungal-loaded CS beads in a 3D skin epidermis coculture model. (A) Schematic representation of the coculture skin wound model used for C. auris inoculation and associated treatment. (B) The colony forming equivalents (CFE) for C. auris colonized on the tissue were determined following DNA extraction and qPCR. (C) RNA was extracted from the tissue and the transcriptional gene response in the host was determined using a RT^2^ profiler array containing genes associated with inflammation and fungal recognition. Significant reductions shown as follows: **, *P* < 0.01; ***, *P* < 0.001.

The final part of this study served to assess the efficacy of the antifungal-loaded CS beads in reducing C. auris fungal load within a skin epidermis coculture model. The experimental setup is highlighted in Fig. 3A. Results showed that treatment with AMB completely diminished the fungal burden in the wounded tissue to levels below qPCR detection (∼1 × 10^3^; ***, *P* < 0.001). CSP was effective in reducing the fungal load 2-fold from ∼1.2 × 10^4^ colony forming equivalents (CFE)/tissue in controls to ∼0.6 × 10^4^ CFE/tissue following treatment (***, *P* < 0.001). FLZ treatment did not change the overall levels of C. auris colonized within the tissue (Fig. 3B). Interestingly, gene expression profiling of the host tissue revealed little reduction in the inflammatory response following treatment with the antifungals, particularly with CSP, which drove elevated expression in genes, such as *TNF*, *IL-6*, *CSF2*, *CSF3*, *TLR4*, *CLEC7A*, and *CAMP* (Fig. 3C).

Candida auris has gained a considerable amount of scientific attention since its emergence in 2009 (1). The organism has a worrying antifungal resistance profile to FLZ, with some isolates exhibiting resistant to AMB and CSP (2, 11). Given the organisms ability to persist in a variety of clinical sites and survive under harsh environmental conditions, it remains a global problem in intensive care units for acquired nosocomial infections. It has been shown recently using clinical studies and *in vivo* and *in vitro* models that C. auris will readily colonize skin (4–6, 12), thus posing a concern for patients with unhealing wounds. Here, we have shown that antifungal-loaded CS beads have the potential to combat C. auris infection by alleviating viability and fungal load *in vitro*.

Firstly, in line with previous observations (13, 14), all three antifungal CS beads exhibited a sustained release of drug over the course of the broth dilution experiment with comparable susceptibility profiles across 7 days. As expected, C. auris was more susceptible to AMB and CSP, both under planktonic and sessile conditions, than FLZ, which has commonly been reported in various C. auris isolates; it is proposed that mutations in genes that participate in the ergosterol synthesis pathway (e.g., *ERG11*) and drug efflux pumps (e.g., *CDR1*) serve to increase FLZ tolerance (15, 16). Nonetheless, CSP- and AMB-loaded CS beads were suitable in reducing metabolic activity and biomass of developing C. auris biofilms grown over 5 days, suggesting that the biological activity of the eluted antifungal remained high even during coincubation with the fungi.

The above observations were strengthened by clear reductions in fungal load following treatments with CSP and AMB, as confirmed with qPCR and SEM imagery. Taken together, these results are suggestive that such CS beads could provide an interesting proposition in wound care, with particular emphasis on antifungal delivery. Thus, to mimic the packing of infected tissue with loaded CS beads, an *in vitro* 3D wounded skin epidermis model was used as previously described (12). In this previous publication, we demonstrated that the C. auris NCPF 8978 strain used in this study was proinflammatory to wounded skin epidermis. Here, although overall fungal load was significantly reduced in tissues packed with CSP- and AMB-loaded CS beads, the inflammatory profile of the tissue remained largely unchanged when compared to control-treated samples. Depending on the treatment modality, these observations may be that the fungal organism, or its constituents, are still present within the tissue model, thereby recognized by the host, which responds accordingly. Alternatively, it may be due to toxicity of the drugs to the host, which is not unheard of. For example, AMB, which was effective in significantly reducing fungal load, still gave rise to a comparable immune response to the controls. Indeed, concentrations of >5 μg/mL have been shown to be highly cytotoxic to host cells (17), which are far lower than the doses packed into the CS beads. Regarding the elevated host response in CSP-loaded antifungal beads, it has recently been shown that CSP treatment increases chitin and glucan exposure on different *Candida* species, which led to elevated recognition and phagocytosis by macrophages (18). Thus, the drug may induce structural changes to the persistent fungi still on the tissue, which consequentially leads to an increased immune response. Nevertheless, it was reassuring that CS control beads did not alter the inflammatory response within the tissue, with results in gene expression comparable with our previous study using C. auris only (12).

In conclusion, we have provided a proof-of-concept study that has investigated the function of antifungal-packed CS beads in controlling C. auris biofilm formation and host-related pathogenicity. This study has been developed to form a preclinical investigation into the efficacy of antifungal-loaded CS beads: future work merits consideration of assessing the effects of the CS beads *in vivo*. Additionally, as a potential use for CS beads is being studied for wound care and infection control (9, 10, 19), future work must consider the CS bead-host interface, particularly given the importance of the immune response in driving wound healing and repair.

### Microbial growth.

Candida auris NCPF 8978 used throughout this study was grown as previously described (12).

### Preparation of Stimulan Rapid Cure calcium sulfate beads.

Stimulan Rapid Cure calcium sulfate beads were prepared as previously described (13). Beads were formed to a size of 6 mm or 3 mm, dependent on experimental set up. The antifungal loads per bead were determined to be 0.56 mg of AMB, 0.38 mg of CSP, and 0.79 mg of FLZ.

### Planktonic susceptibility testing.

To assess antifungal susceptibility, planktonic broth microdilution tests were performed as a surrogate marker of drug release as described elsewhere (13).

### Biofilm susceptibility testing.

To test the sessile susceptibility of C. auris to the antifungal-loaded CS beads, biofilms were formed as discussed in a previous publication (13). In brief, metabolic activity was assessed using an XTT (2,3-bis-(2-methoxy-4-nitro-5-sulfophenyl)-2H-tetrazolium-5-carboxanilide salt) assay. The XTT reagent was removed, and biofilms were left to dry to assess biomass using the crystal violet (CV) assay.

### Quantitative PCR.

Treated biofilms were formed on 13-mm Thermanox coverslips and DNA extracted for SYBR GreenER-based qPCR (13). For this, 18S primers were used with the following thermal profile: 50°C for 2 min, denaturation stage of 95°C for 10 min, and then 40 cycles of 95°C for 3 s and 60°C for 15 s using the StepOne real-time PCR system. Colony forming equivalent (CFE) counts were determined using a standard curve of known fungal CFU ranging from 1 × 10^3^ to 1 × 10^8^ CFU/mL.

### Scanning electron microscopy.

Additional treated biofilms were used for scanning electron microscopy (SEM). In brief, coverslips containing the biofilms were removed and initially fixed and then further processed for SEM imaging as described previously (13, 20). Biofilms were visualized using a JEOL JSM-6400 scanning electron microscope (JEOL Ltd, Hertfordshire, UK).

### Skin epidermis coculture system.

C. auris was inoculated on a wounded 3D skin epidermis model (SkinEthic; Episkin, Lyon, France) with CS beads. The model set up was created as previously described (12), with small amendments as followed. At the same time as the addition of C. auris, 5- by 3-mm CS beads loaded with AMB, CSP, and FLZ, were also added to tissue samples to assess the antifungal effect of CS beads in this coculture system. The cocultured model was incubated for 24 h at 5% CO_2_, 37°C.

Following coculture, DNA and RNA were extracted from the tissue to assess fungal load and host gene expression using the QIAamp DNA minikit with bead beating and the Qiagen RNeasy minikit (Qiagen Ltd, Crawley, UK), respectively. Fungal load was assessed using qPCR as above. For the host gene response, RNA was converted to cDNA before a custom RT^2^ profiler array was used to assess changes in inflammatory gene markers. Detailed methods for this are discussed elsewhere (12).

### Statistical analysis.

Statistical analyses and graph production were performed using GraphPad Prism (version 8.4.3; GraphPad Software Inc., La Jolla, CA). One-way analysis of variance (ANOVA) with Tukey’s post-test was used to assess differences in antifungal efficacies. For CFU and CFE counts, data was log transformed before analysis. Statistical significance was achieved if *P* was <0.01.

## References

[1] Kim MN, Shin JH, Sung H, Lee K, Kim EC, Ryoo N, Lee JS, Jung SI, Park KH, Kee SJ, Kim SH, Shin MG, Suh SP, Ryang DW. 2009. Candida haemulonii and closely related species at 5 university hospitals in Korea: identification, antifungal susceptibility, and clinical features. Clin Infect Dis 48:e57–e61. 10.1086/597108. 19193113

[2] Kean R, Ramage G. 2019. Combined antifungal resistance and biofilm tolerance: the global threat of Candida auris. mSphere 4:e00458-19. 10.1128/mSphere.00458-19. 31366705 PMC6669339

[3] Short B, Brown J, Delaney C, Sherry L, Williams C, Ramage G, Kean R. 2019. Candida auris exhibits resilient biofilm characteristics in vitro: implications for environmental persistence. J Hosp Infect 103:92–96. 10.1016/j.jhin.2019.06.006. 31226270

[4] Horton MV, Johnson CJ, Kernien JF, Patel TD, Lam BC, Cheong JZA, Meudt JJ, Shanmuganayagam D, Kalan LR, Nett JE. 2020. Candida auris forms high-burden biofilms in skin niche conditions and on porcine skin. mSphere 5:e00910-19. 10.1128/mSphere.00910-19. 31969479 PMC6977180

[5] Huang X, Hurabielle C, Drummond RA, Bouladoux N, Desai JV, Sim CK, Belkaid Y, Lionakis MS, Segre JA. 2021. Murine model of colonization with fungal pathogen Candida auris to explore skin tropism, host risk factors and therapeutic strategies. Cell Host Microbe 29:210–221. 10.1016/j.chom.2020.12.002. 33385336 PMC7878403

[6] Proctor DM, Dangana T, Sexton DJ, Fukuda C, Yelin RD, Stanley M, Bell PB, Baskaran S, Deming C, Chen Q, Conlan S, Park M, Mullikin J, Thomas J, Young A, Bouffard G, Barnabas B, Brooks S, Han J, Ho S-l, Kim J, Legaspi R, Maduro Q, Marfani H, Montemayor C, Riebow N, Schandler K, Schmidt B, Sison C, Stantripop M, Black S, Dekhtyar M, Masiello C, McDowell J, Thomas P, Vemulapalli M, Welsh RM, Vallabhaneni S, Chiller T, Forsberg K, Black SR, Pacilli M, Kong HH, Lin MY, Schoeny ME, Litvintseva AP, Segre JA, Hayden MK, NISC Comparative Sequencing Program. 2021. Integrated genomic, epidemiologic investigation of Candida auris skin colonization in a skilled nursing facility. Nat Med 27:1401–1409. 10.1038/s41591-021-01383-w. 34155414 PMC9396956

[7] McConoughey SJ, Howlin RP, Wiseman J, Stoodley P, Calhoun JH. 2015. Comparing PMMA and calcium sulfate as carriers for the local delivery of antibiotics to infected surgical sites. J Biomed Mater Res B Appl Biomater 103:870–877. 10.1002/jbm.b.33247. 25142105

[8] Abosala A, Ali M. 2020. The use of calcium sulphate beads in periprosthetic joint infection, a systematic review. J Bone Jt Infect 5:43–49. 10.7150/jbji.41743. 32117689 PMC7045528

[9] Dekker AP, Uzoho C, Scammell B. 2019. Do antibiotic-impregnated calcium sulfate beads improve the healing of neuropathic foot ulcers with osteomyelitis undergoing surgical debridement? Wounds 31:145–150. 31184595

[10] Patil P, Singh R, Agarwal A, Wadhwa R, Bal A, Vaidya S. 2021. Diabetic foot ulcers and osteomyelitis: use of biodegradable calcium sulfate beads impregnated with antibiotics for treatment of multidrug-resistant organisms. Wounds 33:70–76. 33793412

[11] Chaabane F, Graf A, Jequier L, Coste AT. 2019. Review on antifungal resistance mechanisms in the emerging pathogen Candida auris. Front Microbiol 10:2788. 10.3389/fmicb.2019.02788. 31849919 PMC6896226

[12] Brown JL, Delaney C, Short B, Butcher MC, McKloud E, Williams C, Kean R, Ramage G. 2020. Candida auris phenotypic heterogeneity determines pathogenicity in vitro. mSphere 5:e00371-20. 10.1128/mSphere.00371-20. 32581078 PMC7316489

[13] Butcher MC, Brown JL, Hansom D, Wilson-van Os R, Delury C, Laycock PA, Ramage G. 2021. Assessing the bioactive profile of antifungal-loaded calcium sulfate against fungal biofilms. Antimicrob Agents Chemother 65:e02551-20. 10.1128/AAC.02551-20. 33753336 PMC8316021

[14] Aiken SS, Cooper JJ, Florance H, Robinson MT, Michell S. 2015. Local release of antibiotics for surgical site infection management using high-purity calcium sulfate: an in vitro elution study. Surg Infect (Larchmt) 16:54–61. 10.1089/sur.2013.162. 25148101 PMC4363816

[15] Healey KR, Kordalewska M, Jimenez Ortigosa C, Singh A, Berrio I, Chowdhary A, Perlin DS. 2018. Limited ERG11 mutations identified in isolates of Candida auris directly contribute to reduced azole susceptibility. Antimicrob Agents Chemother 62:e01427-18. 10.1128/AAC.01427-18. PMC6153782 30082281

[16] Bhattacharya S, Holowka T, Orner EP, Fries BC. 2019. Gene duplication associated with increased fluconazole tolerance in Candida auris cells of advanced generational age. Sci Rep 9:5052. 10.1038/s41598-019-41513-6. 30911079 PMC6434143

[17] Grela E, Piet M, Luchowski R, Grudzinski W, Paduch R, Gruszecki WI. 2018. Imaging of human cells exposed to an antifungal antibiotic amphotericin B reveals the mechanisms associated with the drug toxicity and cell defence. Sci Rep 8:14067. 10.1038/s41598-018-32301-9. 30218099 PMC6138690

[18] Walker LA, Munro CA. 2020. Caspofungin induced cell wall changes of Candida Species influences macrophage interactions. Front Cell Infect Microbiol 10:164. 10.3389/fcimb.2020.00164. 32528900 PMC7247809

[19] Menon A, Soman R, Rodrigues C, Phadke S, Agashe VM. 2018. Careful interpretation of the wound status is needed with use of antibiotic impregnated biodegradable synthetic pure calcium sulfate beads: series of 39 cases. J Bone Jt Infect 3:87–93. 10.7150/jbji.22684. 29922571 PMC6004684

[20] Erlandsen SL, Kristich CJ, Dunny GM, Wells CL. 2004. High-resolution visualization of the microbial glycocalyx with low-voltage scanning electron microscopy: dependence on cationic dyes. J Histochem Cytochem 52:1427–1435. 10.1369/jhc.4A6428.2004. 15505337 PMC3957825

